# Focal Adhesion Kinase (FAK) Mediates the Induction of Pro-Oncogenic and Fibrogenic Phenotypes in Hepatitis C Virus (HCV)-Infected Cells

**DOI:** 10.1371/journal.pone.0044147

**Published:** 2012-08-28

**Authors:** Anna Alisi, Mario Arciello, Stefania Petrini, Beatrice Conti, Gabriele Missale, Clara Balsano

**Affiliations:** 1 Liver Research Unit, Confocal Microscopy Facility of “Bambino Gesù” Children’s Hospital, IRCCS, Rome, Italy; 2 Laboratory of Molecular Virology and Oncology, A. Cesalpino Foundation, University of Rome “La Sapienza”, Rome, Italy; 3 Department of Internal Medicine, University of L’Aquila, L’Aquila, Italy; 4 Confocal Microscopy Facility of “Bambino Gesù” Children’s Hospital, IRCCS, Rome, Italy; 5 Unit of Infectious Diseases and Hepatology, Azienda Ospedaliero – Universitaria di Parma, Parma, Italy; 6 IBPM (Institute of Biology and Molecular Pathology), Rome, Italy; Ospedale Pediatrico Bambino Gesù, Italy

## Abstract

Hepatitis C Virus (HCV) infection is one of the most common etiological factors involved in fibrosis development and its progression to hepatocellular carcinoma (HCC). The pivotal role of hepatic stellate cells (HCSs) and extracellular matrix (ECM) in fibrogenesis is now certainly accepted, while the network of molecular interactions connecting HCV is emerging as a master regulator of several biological processes including proliferation, inflammation, cytoskeleton and ECM remodeling. In this study, the effects of HCV proteins expression on liver cancer cells, both pro-invasive and pro-fibrogenic phenotypes were explored. As a model of HCV infection, we used permissive Huh7.5.1 hepatoma cells infected with JFH1-derived ccHCV. Conditioned medium from these cells was used to stimulate LX-2 cells, a line of HSCs. We found that the HCV infection of Huh7.5.1 cells decreased adhesion, increased migration and caused the delocalization of alpha-actinin from plasma membrane to cytoplasm and increased expression levels of paxillin. The treatment of LX-2 cells, with conditioned medium from HCV-infected Huh7.5.1 cells, caused an increase in cell proliferation, expression of alpha-smooth muscle actin, hyaluronic acid release and apoptosis rate measured as cleaved poly ADP-ribose polymerase (PARP). These effects were accompanied in Huh7.5.1 cells by an HCV-dependent increasing of FAK activation that physically interacts with phosphorylated paxillin and alpha-actinin, and a rising of tumor necrosis factor alpha production/release. Silencing of FAK by siRNA reverted all effects of HCV infection, both those directed on Huh7.5.1 cells, and those indirect effects on the LX-2 cells. Moreover and interestingly, FAK inhibition enhances apoptosis in HCV-conditioned LX-2 cells. In conclusion, our findings demonstrate that HCV, through FAK activation, may promote cytoskeletal reorganization and a pro-oncogenic phenotype in hepatocyte-like cells, and a fibrogenic phenotype in HSCs.

## Introduction

Hepatitis C Virus (HCV) infection affects approximately 170 million people worldwide, increasing the risk of cirrhosis and hepatocellular carcinoma (HCC), which represents the fifth most frequent cancer in the world and the third most frequent cause of tumor-related death [1], [2]. Several studies have been performed in *in vitro* artificial models to explore the potential hepatocarcinogenic effects of *in vivo* HCV infection. In particular, HCV proteins, both directly and indirectly, may interfere with the genes/proteins that regulate fibrogenesis and pro-oncogenic effects [3]–[9].

During the last decade, it has become evident that not only the tumor cell itself, but also the tumor microenvironment plays a major role in the development of HCC. In fact, a direct link between the carcinogenic roles of inflammation, advanced liver fibrosis, epithelial to mesenchymal transition (EMT), tumor invasion and metastasis with microenvironment around the liver cells has been reported [8], [9]. Therefore, HCC pathogenesis results were associated with a progressive loss of cell differentiation, as well as to alterations of cell-extracellular matrix (ECM) functions. ECM is characterized by the constitutive activation of selected cellular signal transduction pathways controlling tissue remodeling; which in turn is strongly associated to the cell cross-talk with the intercellular surrounding microenvironment [10], [11]. The most relevant of these pathways is controlled by focal adhesion kinase (FAK), which is a 125 kDa cytoplasmic tyrosine kinase preferentially localized into cellular focal contacts. FAK is activated by an integrin-mediated engagement and its autophosphorylation at tyrosine 397 in the N-terminal domain is a prerequisite to trigger its activity as a signaling protein within cytoskeleton-associated networks. FAK activation induces tyrosine phosphorylation of multiple cellular proteins, including alpha-actinin and paxillin, which results in signaling cascades able to affect both cell adhesion and spreading [12], [13]. Numerous studies have suggested that FAK is overexpressed in a variety of human tumors, including HCC, and plays an important role in neoplastic transformation and malignant progression [14]–[18]. The role of FAK in HCV-dependent hepatocarcinogenesis is supported by the identification of focal adhesion proteins as HCV potential targets [19]. Up-regulation of FAK in HCCs has been associated with the promotion of portal venous invasion and consequently intra-hepatic metastasis [17], [18]. Furthermore, FAK may influence proliferation and activation of hepatic stellate cells (HSCs), resulting crucial in hepatic fibrogenesis [20], [21]. The activation of HSCs is recognized as a central event in the development of hepatic fibrosis and lastly, cirrhosis. Activated HSCs are primarily responsible for an excess of collagen deposition during liver fibrosis, because they become directly fibrogenic by synthesizing ECM proteins [22]. Moreover, HSCs are located around tumor sinusoids, fibrous septa and the vessels of the capsule, if the latter is present [23].

Here, we emphasize the direct role of FAK as mediator of pro-oncogenic phenotype in HCV-infected hepatocytes and its crucial role as a indirect regulator of fibrogenic induction of HSCs by HCV-dependent paracrine mechanism.

## Results

### HCV Infection Affects Proliferation, Anchorage-independent Growth, Adhesion and Migration of Huh7.5.1 Cells

**Figure 1:**
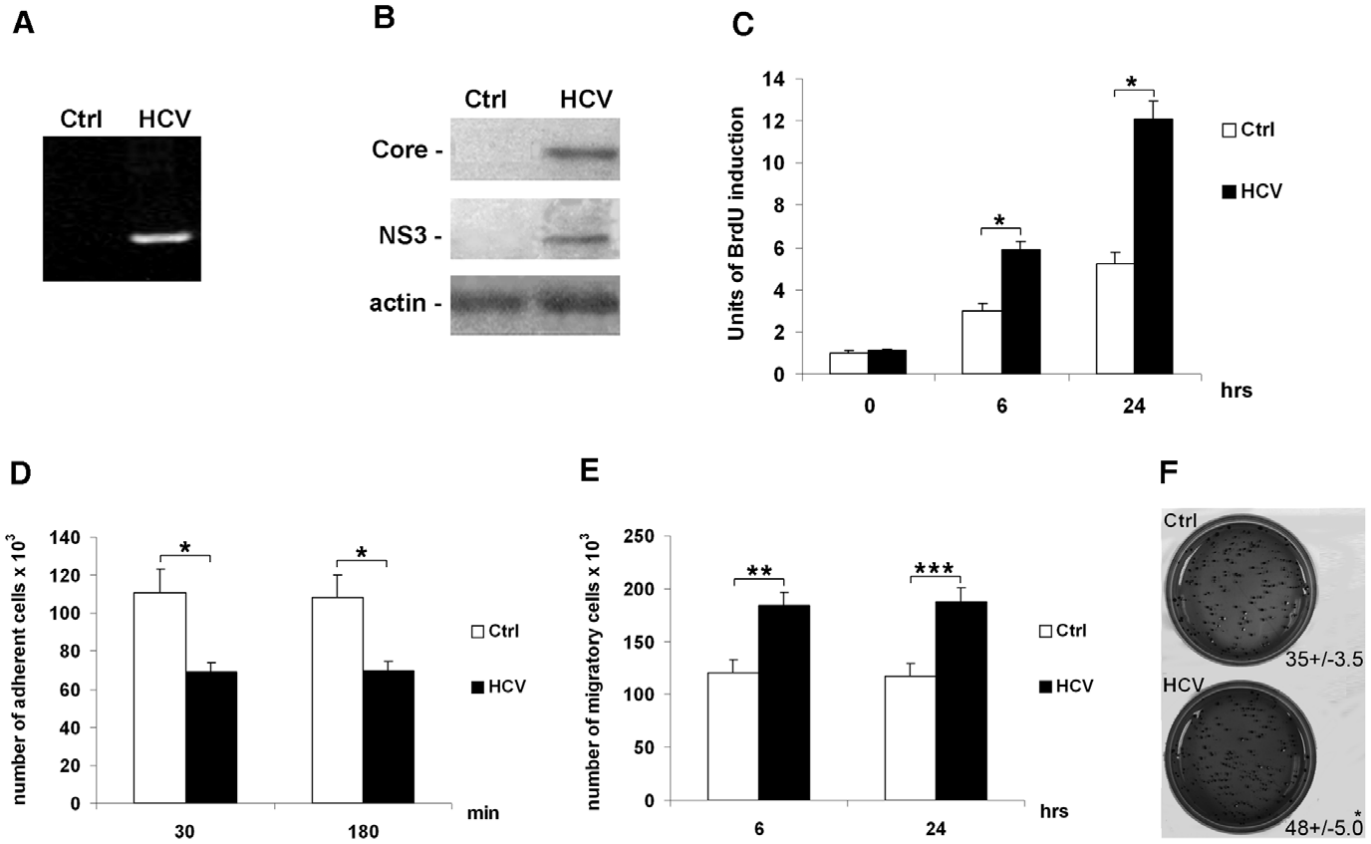
HCV affects proliferation, anchorage-independent growth, adhesion and migration of Huh7.5.1 cells. (**A**) RT-PCR for the 5′UTR in control (Ctrl) and HCV-infected Huh7.5.1 cells (HCV). (**B**) Protein expression levels of HCV core (*upper panel*), HCV NS3 (*middle panel*) and beta-actin (loading control, *lower panel*) in control (Ctrl) and HCV-infected Huh7.5.1 cells (HCV). Immunoblots are representative of at least five independent experiments. (**C**) Proliferation rate was evaluated as incorporation of BrdU performed at three different time points: 0, 6 and 24 hrs. Quantitative data of the analysis of BrdU incorporation was converted in unit of induction with respect to the Ctrl considered as 1. Histograms are the mean value ±SD (*bars*) of five independent experiments. *P<0.001 *versus* Ctrl. (**D**) Adhesion test, and (**E**) cell migration assay. Data reported in the histograms (panels **D** and **E**) were expressed as the number of adherent or migrating cells × 10^3^ compared with controls, considered as 100×10^3^. *Columns*, means of three independent experiments; *bars*, ±S.D. P value was calculated *versus* the control cells: *P<0.001; **P<0.01; ***P<0.05. (F) Anchorage-independent growth was evaluated by colony formation assay in soft agar. After 28 days of incubation; colonies >0.15 mm in size were counted under microscope (×10 magnification). Data is expressed as means±SD (*bars*) of three independent experiments. *P<0.05 *versus* Ctrl.

To investigate whether expression of HCV proteins promotes the acquisition of invasiveness ability in HCC cells, we used an HCV-model system that mimics the *in vivo* HCV infection: JFH1-derived ccHCV cell culture system [24]. Huh7.5.1 cells were infected at multiplicity of infection (MOI) of 0.1. five days post-infection, cells were harvested to perform the RT-PCR for 5′UTR (**Figure 1A**) and the analysis of expression of two HCV proteins: core and NS3 (**Figure 1B**). The two analyzed proteins, well expressed by our HCV infection system, confirmed that JFH1-derived ccHCV works good in Huh7.5.1 cells. Uninfected Huh7.5.1 cells (Ctrl) and HCV-infected Huh7.5.1 cells being five days post-infection (HCV) were plated in 6-well plates and subjected to further analyses. Next, we analyzed the rate of cell proliferation by quantitative analysis of BrdU incorporation at different time points from plating (0, 6, 24 hours). As shown in **Figure 1C**, the HCV-infected cells displayed a significant increase in their proliferation rate with respect to the control cells. These results were also confirmed by long-term effects (48 and 96 hours) on growth rate of uninfected and HCV-infected Huh7.5.1 cells (Figure S1).

The adhesion capability of cells was analyzed by calculating the number of adherent cells after 30 and 180 min from plating. As reported in **Figure 1D**, reduced adhesiveness was observed in HCV-infected cells when compared with uninfected cells. On the other hand, the migration rate, evaluated at 6 and 24 hrs after plating, was significantly increased in HCV-infected cells with respect to the control cells (**Figure 1E**). We, then, looked at the effect of HCV on growth of colonies in soft agar that provides a measure of the cell ability grow in an anchorage-independent manner. We found that HCV-infected Huh7.5.1 cells formed, after 28 days, larger colonies than uninfected cells (**Figure 1F**).

### HCV Infection Affects Intracellular Localization, Expression Levels, and Activities of Focal Adhesion Molecules

Many different cell adhesion molecules and cytoskeletal elements, involved in intercellular and cell-ECM interactions, may regulate adhesion and migration, including paxillin (adaptor), alpha-actinin (structural protein), and FAK (enzyme) [25]–[27]. Not only the protein expression, but also a precise intracellular positioning and activity of these molecules is required in normal cells for the maintenance of their correct biological roles.

**Figure 2:**
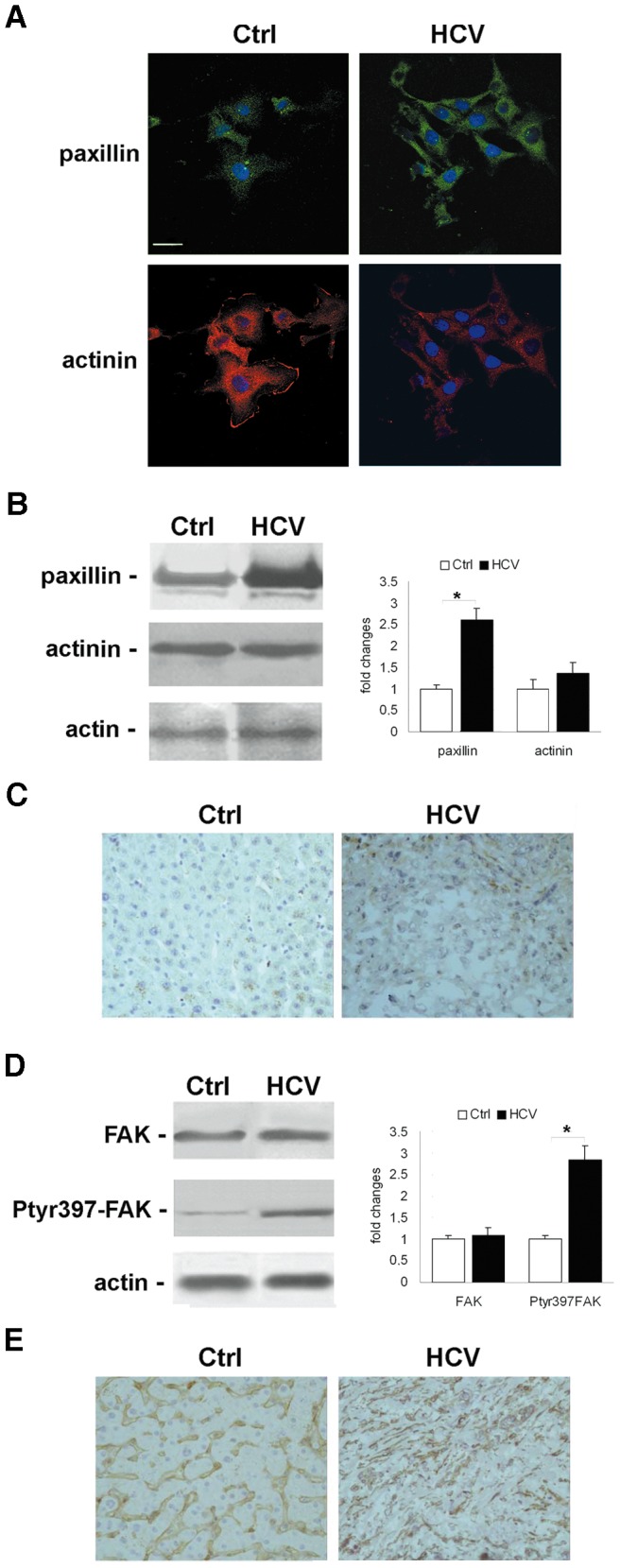
HCV infection affects intracellular localization, expression levels, and activities of focal adhesion molecules. (**A**) Immunofluorescence of paxillin (*green*), and alpha-actinin (*red*) by confocal microscopy in Ctrl and HCV Huh7.5.1 cells after 24 hrs from plating. DAPI (*blue*) was included to stain the nuclei. Magnification bar: 30 µ. (**B**) Paxillin and alpha-actinin protein expression levels observed 24 hrs after plating. (**C**) Immunohistochemical analysis of paxillin expression in HCCs and control livers (×200 magnification). (**D**) Total FAK and tyrosine 397 phosphorylated FAK were observed 24 hrs after plating. Immunoblots are representative of at least four independent experiments. (**E**) Immunohistochemical analysis of tyrosine 397 phosphorylated FAK expression in HCCs and control livers (×200 magnification). In left histograms densitometric analysis is reported as fold changes in protein levels respect to the control cells considered as 1 after normalization against beta-actin (as loading control). *P<0.001 versus Ctrl.

The intracellular localization of paxillin and alpha-actinin was analyzed by confocal laser microscopy 24 hrs after plating. As shown in **Figure 2A**, paxillin appeared diffused throughout the cytoplasm, both by the absence and presence of HCV infection, even if the signal was stronger in the latter. In control cells, alpha-actinin appears localized on both the plasma membrane and the cytoplasm, whereas in HCV-infected cells, it was restricted to the cytoplasm only (**Figure 2A**). Western blot analysis and the densitometric evaluation of bands showed that, while there was a significant increase in the expression levels of paxillin (**Figure 2B**, *upper panel*, and *left histogram*), no relevant changes of alpha-actinin were found in HCV-infected cells (**Figure 2B**, *middle panel*, and *left histogram*). Similarly, we found an increased expression of paxillin in HCV-related HCCs with respect to control livers (**Figure 2C**).

Next, we analyzed the effects of HCV infection on FAK expression and activity after 24 hrs from cell re-plating. A significant increase of tyrosine-397 phosphorylated FAK expression levels was observed in HCV-infected cells (**Figure 2D**, *middle panel* and *left histogram*), whereas no variation of total FAK expression occurred (**Figure 2D**, *upper panel* and *left histogram*). These results were confirmed by the immuno-histochemical analysis of livers from HCV-related HCC patients and control subjects. As shown in **Figure 2E**, we observed an increased expression of tyrosine-397 phosphorylated FAK in HCC due to HCV.

### Conditioned Medium from HCV-infected Cells Induces Proliferation, Invasion, Activation and a Pro-fibrogenic Phenotype in LX-2 Cells

**Figure 3:**
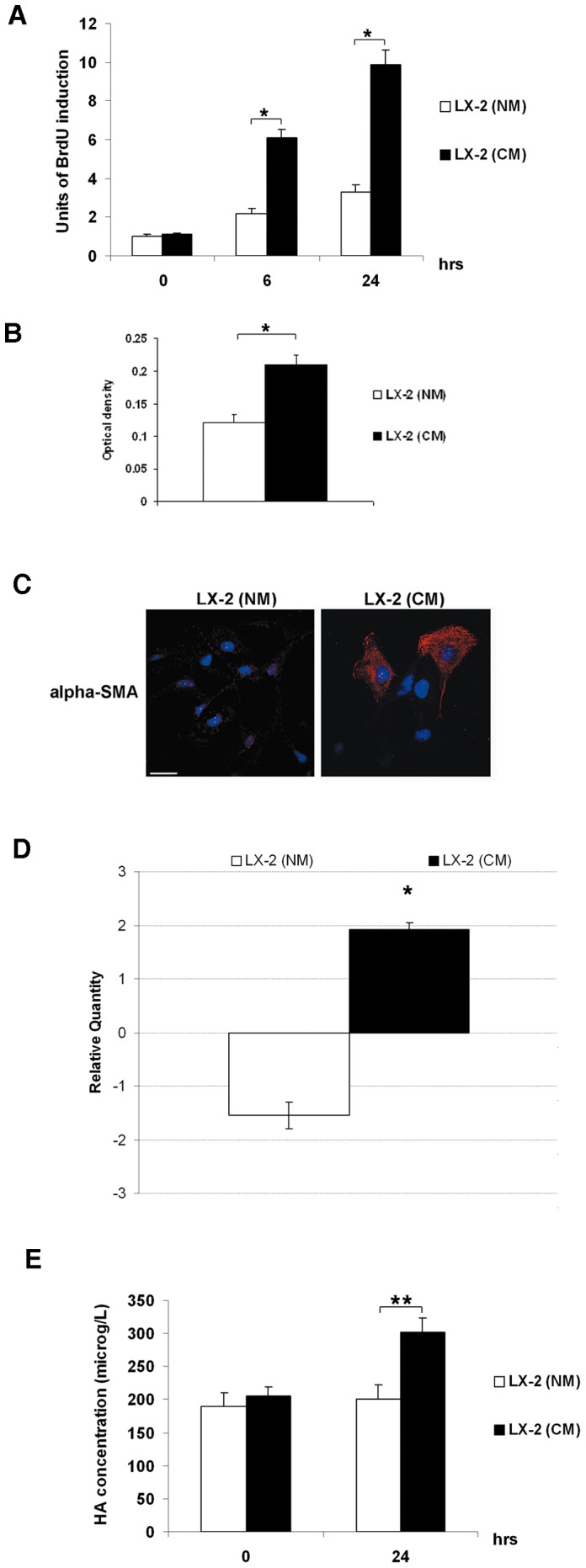
HCV infection indirectly induces proliferation, invasion, activation and a pro-fibrogenic phenotype in LX-2 cells. (A) Proliferation rate of LX-2 (NM) and LX-2 (CM) was evaluated as incorporation of BrdU performed at three different time points: 0, 6 and 24 hrs. Quantitative data of the analysis of BrdU incorporation was converted in unit of induction with respect to the Ctrl considered as 1. Histograms are the mean value ±SD (*bars*) of five independent experiments. *P<0.001 *versus* LX-2 (NM). (**B**) The invasive abilities of LX-2 (NM) and LX-2 (CM) were examined after 24 hrs by chamber assay. Spectrophotometrical analysis (optical density) was employed for quantification of the transmembrane cells. Histogram reports the mean value ±SD (*bars*) of three independent experiments. *P<0.01 *versus* LX-2 (NM). (**C**) Immunofluorescence of alpha-SMA (*red*) by confocal microscopy in LX-2 (NM) and LX-2 (CM) cells after 24 hrs stimulus. DAPI (*blue*) was included to stain the nuclei. Magnification bar: 30 µ. (**D**) Real-time PCR for the expression of alpha-SMA mRNA. The histogram reports the relative quantity of alpha-SMA mRNA normalized for actin. Values between +1 and –1 indicating invariant expression, are not shown. Data is statistically significant (*P≤0.05) for relative quantity greater than ±1.5. (**E**) Concentration of HA by ELISA assay in medium collected at time 0 and after 24 hrs of treatment from LX-2 (NM) and LX-2 (CM) cells. Histograms report the mean values (microg/L of HA) ±SD, (*bars*) of 4 independent experiments. *P<0.001; **P<0.01 *versus* LX-2 (NM).

To determine whether HCV infection may influence proliferation, invasion and activation of HSCs, medium from Huh7.5.1 control cells (normal medium, NM) and HCV-infected Huh7.5.1 cells (conditioned medium, CM) was collected after 72 hrs from re-plating and used in combination (1∶1) with 10% FBS medium, to culture LX-2 cells. Before the addition, the medium was cleared and negativity for HCV was assessed by RT-PCR for 5′UTR. As shown in **Figure 3A**, in contrast to LX-2 plus NM, the LX-2 plus CM displayed a significantly increased proliferation rate after 6 and 24 hrs. Moreover, also LX-2 invasiveness after 24 hrs was significantly enhanced by the treatment with CM **(** **Figure 3B** **)**. Concomitantly, by confocal laser microscopy, we observed an increase in alpha-smooth muscle actin (SMA), a marker of myofibroblast differentiation (**Figure 3C**). This last result is supported by the increase of alpha-SMA mRNA expression in LX-2 plus CM (**Figure 3D**).

Finally, after 24 hrs, the medium from LX-2 cells was collected and analyzed by ELISA assay to determine the release of hyaluronic acid (HA). As shown in **Figure 3E**, the significant increase of HA released in the medium of LX-2 plus CM with respect to LX-2 plus NM, was an additional indirect effect of the HCV infection.

### Silencing of FAK Counteracts HCV-dependent Direct Effects on Huh7.5.1 Cells

**Figure 4:**
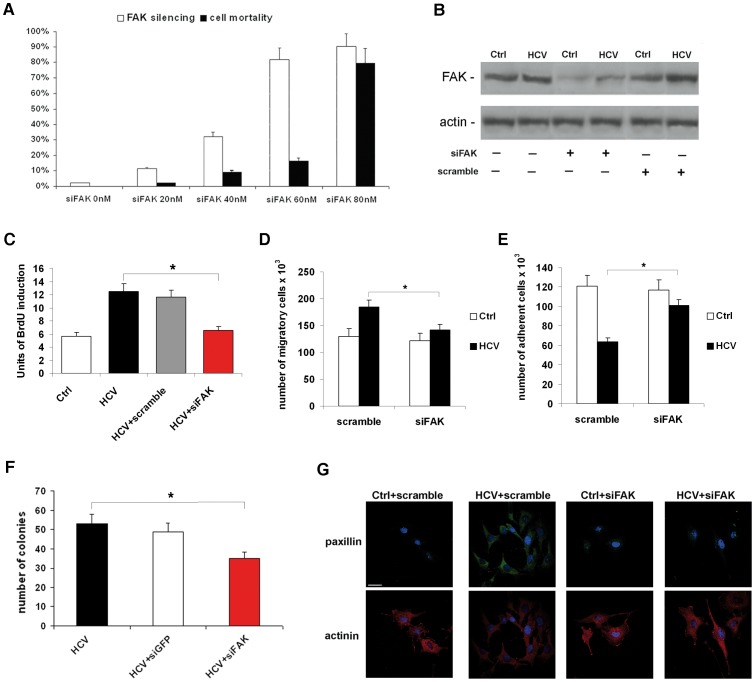
FAK siRNA counteracts HCV-dependent direct effects on Huh7.5.1 cells. (**A**) Percentage of cell mortality and FAK silencing after 24 hrs from treatment of HCV-infected cells with different amounts of FAK siRNA with respect to the control siRNA considered as 0. Histograms represent % Trypan Blue positive cells and % of inhibition of FAK protein expression ± SD (*bars*). *P<0.001. (**B**) Protein expression levels of total FAK 24 hrs after siRNA transfection. Immunoblots are representative of at least four independent experiments. (**C**) Proliferation rate was evaluated as incorporation of BrdU performed after 24 hrs from siRNA transfection. Quantitative data of the analysis of BrdU incorporation was converted in unit of induction with respect to the Ctrl considered as 1. Histograms are the mean value ±SD (*bars*) of five independent experiments. *P<0.001. (**D**) Cell migration assay, and (**E**) adhesion test. Data reported in the histograms (panels c and d) is expressed as the number of adherent or migrating cells compared with controls, considered as 100×0^3^. *Columns*, means of three independent experiments ±SD (*bars*). *P<0.001. (**F**) Anchorage-independent growth was evaluated by colony formation assay in soft agar. After 28 days of incubation, colonies >0.15 mm in size were counted under microscope (×10 magnification). Data reported in the histogram is expressed as means ±SD (*bars*) of three independent experiments. *P<0.01 *versus* Ctrl. (**G**) Immunofluorescence of paxillin (*green*), and alpha-actinin (*red*) by confocal microscopy in Ctrl and HCV Huh7.5.1 cells after 24 hrs from siRNA transfection. DAPI (*blue*) was included to stain the nuclei. Magnification bar: 30 µ.

As reported above, integrin-mediated recruitment of FAK and its consequent activation by autophosphorylation in tyrosine-397 residue is the core of the signaling network that controls focal adhesion molecules [25]–[27]. Thus, to clearly elucidate the role of FAK on HCV-dependent deregulation of focal adhesion molecules, we silenced FAK through specific siRNA in HCV-infected Huh7.5.1 cells. Preliminary western blot experiments showed that the expression of FAK protein was dramatically reduced in HCV-infected cells after 24 hrs from FAK siRNA transfection (concentrations ranging from 20 to 80 nM) (**Figure 4A**, *white columns*). Treatment with 80 nM FAK siRNA caused a reduction of FAK protein expression of up to 90%. Unfortunately, a concomitant dramatic reduction (80%) of cell viability was also observed (**Figure 4A**, *black columns*). On the other hand, using 60 nM FAK siRNA, the protein expression of FAK was reduced by up to 70%, without relevant effects on cell viability (16% reduction) (**Figures 4A**). Therefore, this concentration was used for all subsequent experiments of silencing. To verify the specific silencing, western blotting with 60 nM scramble as control siRNA was performed (**Figure 4B**).

We first explored the effect of FAK siRNA on the pro-proliferative and pro-invasive effects induced by HCV infection in Huh7.5.1 cells. As shown in **Figures 4C, D**, the treatment with FAK siRNA completely reversed the HCV-dependent induction of DNA synthesis and migration after 24 hrs from silencing. The adhesion capability was assayed 180 min from re-plating HCV-infected and uninfected cells transfected with control and FAK siRNAs, 24 hrs before (**Figure 4E**). FAK silencing was able to restore in HCV-infected cells, values of adhesiveness comparable with uninfected cells. FAK silencing also inhibits the HCV-dependent anchorage-independent growth in soft agar after 28 days of culture (**Figure 4F**).

Furthermore, FAK siRNA was able to abolish the HCV-dependent effects on paxillin and alpha-actinin, too. In fact, the amount of paxillin came back to the control values, and alpha-actinin re-localized on the plasma membrane in HCV infected cells (**Figure 4G** and Figure S2).

**Figure 5:**
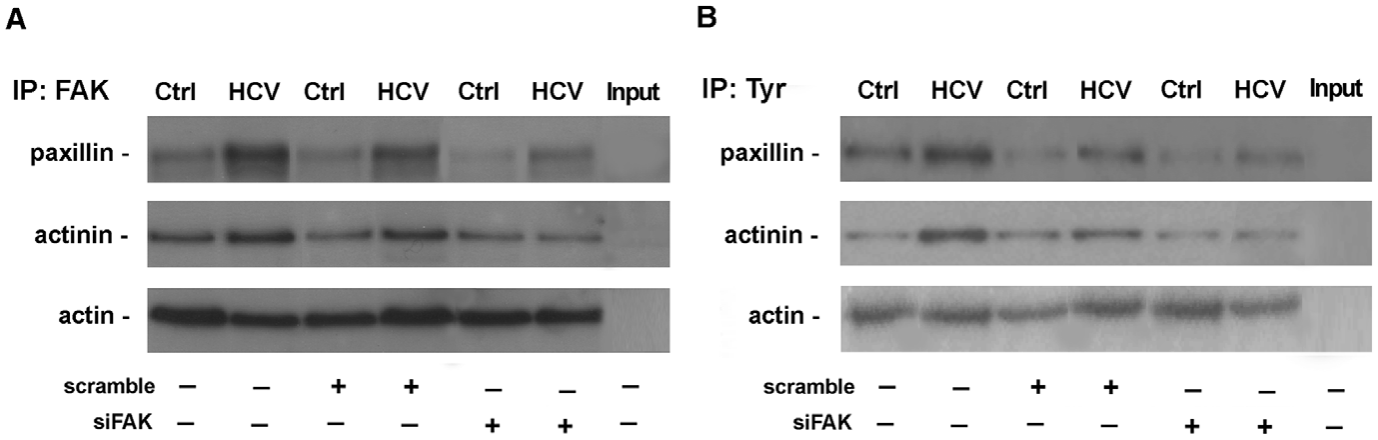
HCV infection promotes physical interaction between FAK and phosphorylated paxillin and alpha-actinin. (**A**) Immunoprecipitation analysis of FAK immunocomplexes in Ctrl and HCV Huh7.5.1 cells after 24 hrs from scramble or siFAK transfection. Western blotting of paxillin (*upper panel*) and alpha-actinin (*middle panel*) was shown. Actin was used as loading control of whole extracts (*lower panel*). Input proteins are reported in the last lane. All blots are representative of three independent experiments. (**B**) Immunoprecipitation analysis of tyrosine phosphorylation of paxillin (*upper panel*) and alpha-actinin (*middle panel*) in Ctrl and HCV Huh7.5.1 cells after 24 hrs from scramble or siFAK transfection. Actin was used as loading control of whole extracts (*lower panel*). Input proteins are reported in the last lane. All blots are representative of three independent experiments.

FAK protein presents a C-terminal region containing 100 residue sequences, designated as focal adhesion targeting, enriched of protein-protein interaction sites [26]. Thus, we analyzed FAK direct physical interaction with paxillin and alpha-actinin. Uninfected and HCV-infected Huh7.5.1 cells, subjected to transfection with control siRNA or siFAK, were lysed and immunoprecipitated with a mouse monoclonal antibody against FAK. FAK immunocomplexes were then assayed for the expression of paxillin and alpha-actinin. As shown in **Figure 5A**, FAK in HCV-infected cells formed complexes, either with paxillin and/or alpha-actinin inhibited by FAK silencing. Furthermore, paxillin and alpha-actinin colocalized in HCV-infected Huh7.5.1 and newly this effect was hampered by siFAK (Figure S3). Paxillin and alpha-actinin are subjected to tyrosine phosphorylation by activated FAK [26], [28]. Noteworthy, immunoprecipitation experiments coupled with western blot analyses (**Figure 5B**) revealed that the HCV infection induced, increased the expression of tyrosine phosphorylated forms of paxillin and alpha-actinin, that were abolished by FAK silencing.

### Silencing of FAK Counteracts HCV-dependent Indirect Effects on LX-2 Cells

**Figure 6:**
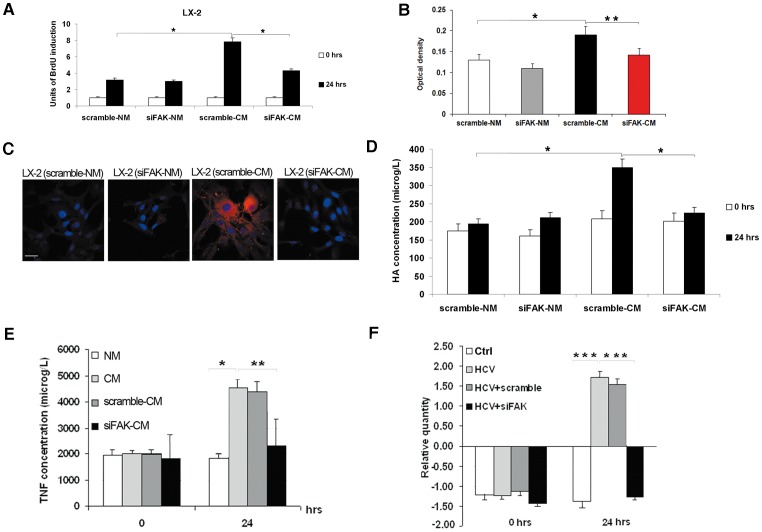
FAK siRNA counteracts HCV-dependent indirect effects on LX-2 cells. (**A**) Proliferation rate of LX-2 treated with scramble-NM, siFAK-NM, scramble-CM and siFAK-CM was evaluated as incorporation of BrdU performed at three different time points: 0 and 24 hrs. Quantitative data of BrdU incorporation was converted in unit of induction with respect to the Ctrl considered as 1. Histograms are the mean value ±SD (*bars*) of five independent experiments. *P<0.001 *versus* Ctrl. (**B**) The invasive abilities of LX-2 treated with scramble-NM, siFAK-NM, scramble-CM and siFAK-CM were examined after hrs by chamber assay. Spectrophotometrical analysis (optical density) was employed for quantification of the transmembrane cells. Data reported in the histogram is the mean value ±SD (*bars*) of three independent experiments. *P<0.01 *versus* scramble-NM; *P<0.05 *versus* scramble-CM. Immunofluorescence of alpha-SMA (*red*) by confocal microscopy in LX-2 treated with scramble-NM, siFAK-NM, scramble-CM and siFAK-CM after 24 hrs of conditioning. DAPI (*blue*) was included to stain the nuclei. Magnification bar: 30 µ. (**C**) Concentration of HA by ELISA assay in medium collected at time 0 and after 24 hrs of treatment of LX-2 with scramble-NM, siFAK-NM, scramble-CM and siFAK-CM. Histogram reports the mean values (microg/L of HA)±SD, (*bars*) of 4 independent experiments. *P<0.001 *versus* Ctrl. (**D**) Concentration of TNF-alpha by ELISA assay in medium collected at time 0 and after 24 hrs from transfection of Hhu7.5.1 cells with scramble and siFAK. Histograms report the mean values (microg/L of TNF-alpha) ±SD, (*bars*) of 4 independent experiments. *P<0.001. (**E**) Real-time PCR for the expression of TNF-alpha mRNA. The histogram reports the relative quantity of TNF-alpha mRNA normalized for actin. Values between +1 and –1 indicating invariant expression, are not shown. Data is statistically significant (*P≤0.05) for relative quantity greater than ±1.5.

FAK phosphorylation can directly promote the proliferation and collagen synthesis of HSCs [29]. We hypothesized that FAK activation in hepatocytes might also indirectly mediate the proliferation, invasion and activation of HSCs. In order to verify our hypothesis, we used media from scramble-transfected (scramble-NM) and siFAK-transfected (siFAK-NM) uninfected Huh7.5.1 cells, or from scramble-transfected (scramble-CM) and siFAK-transfected (siFAK-CM) HCV-infected Huh7.5.1 cells to stimulate LX-2 cells for 24 hrs. As shown in **Figure 6A**, the treatment with siFAK-CM caused a marked decrease in the LX-2 cell proliferation rate, when compared in respect to cells treated with scramble-CM. Noteworthy, the silencing of FAK was also able to remove the indirect pro-invasive effect of HCV in LX-2 cells (**Figure 6B**).

Furthermore, immunofluorescence by confocal laser microscopy showed that FAK silencing indirectly abolished the above reported effect of CM on alpha-SMA in LX-2 (**Figure 6C** and Figure S4). In the same manner, the release of HA from siFAK-CM treated LX-2 with respect to siFAK-NM treated LX-2 was drastically reduced (**Figure 6D**).

Recently, the pivotal role of FAK signaling in regulating cytokine production and release has been reported [30], [31]. Among cytokines, TNF-alpha is a relevant mediator of proliferation and activation of HSCs, and it is also a down-stream factor of HCV infection [32]. Therefore, we hypothesized that the HCV infection could directly increase the production and release of TNF-alpha by hepatocytes, which in turn could promote the indirect effects of HCV virus on HSCs. This possibility was explored by evaluating levels of TNF-alpha released in CM, NM, scramble-CM and siFAK-CM. As shown in **Figure 6E**, medium from HCV-infected cells contained high levels of TNF-alpha that were affected by FAK silencing. This enrichment in circulating levels of TNF-alpha released in CM was explained by an increased transcription of TNF-alpha mRNA in HCV-infected Huh7.5.1 cells. FAK silencing was able to completely revert HCV-dependent TNF-alpha mRNA overexpression (**Figure 6F**).

### Silencing of FAK Enhance HCV-dependent Pro-apoptotic Effects on LX-2 Cells

**Figure 7:**
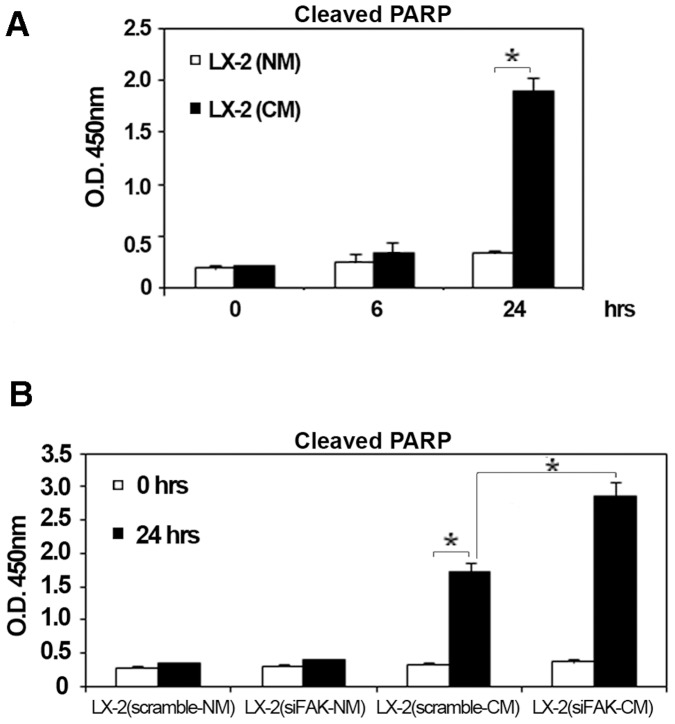
FAK siRNA enhance apoptotic response in HCV conditioned LX-2 cells. (**A**) The histogram reports O.D. at 450 nm ±SD (*bars*) that provides a measure of cleaved PARP after 0, 6 and 24 hrs of LX-2 conditioning with NM and CM. *P<0.001. (**B**) The histogram reports O.D. at 450nm ±SD (*bars*) that provides a measure of PARP cleavage after 0 and 24 hrs of LX-2 conditioning with scramble-NM and siFAK-NM, scramble-CM and siFAK-CM. *P<0.001.

As observed in other cells, HSCs proliferation, activation and differentiation during fibrogenesis is counterbalanced by an increased apoptosis that has been considered a critical process in the natural resolution of liver fibrosis [33]. Therefore, we analyzed the indirect effect of HCV on HSC apoptosis measured as levels of cleave poly ADP-ribose polymerase (PARP). A significant increase of PARP was observed in LX-2 plus CM cells, compared to LX-2 plus NM, after 24 hrs of treatment (**Figure 7A**). In retinal endothelial cells, the FAK pathway cooperates with p38MAPK to maintain cell homeostasis, and the inhibition of these two pathways induces the increment of caspase-3 activation and PARP cleavage, leading to apoptosis [34]. Accordingly, the analysis of cleaved PARP at 24 hrs in LX-2 treated with scramble-CM and siFAK-CM from HCV-infected Huh7.5.1 demonstrated that FAK silencing caused further increased levels of the cleaved PARP in LX-2 under HCV infection (**Figure 7B**)**.**

## Discussion

HCV infection exerts pro-oncogenic and pro-fibrotic effects, maybe interfering with relevant biological cellular processes [2], [3]. Moreover, HCV-dependent liver damage involves several indirect mechanisms, including chronic inflammation, fibrosis, oxidative stress and their physiopathological consequences. Thus, HCV infection may exert differential effects on liver cell populations: i.e. inflammatory response of macrophages, the activation and proliferation of mesenchymal cell populations (HSCs), and the capacity to induce hepatocyte transformation [35]–[38]. In particular, during HCV infection, disturbances of the equilibrium between parenchyma and ECM, leading to a disproportionate increase in the deposition of the newly formed connective tissue components (fibrosis), is a common sequel of chronic active liver diseases with serious clinical consequences, such as cirrhosis and HCC development [39]. Significant progress has been made in recent years in the analysis of the structural composition of ECM in normal and fibrotic liver and in the dissection of the molecular and cellular mechanisms involved in ECM alterations [40]. However, up to now, no one has considered the possible existence of a direct link between the HCV-related ECM alterations, hepatic fibrosis and HCC occurrence. On these grounds, we investigated the effect of HCV infection in the activity and expression of principal proteins involved in intercellular and cell-ECM interactions.

We observed that the HCV infection of Huh7.5.1 cells decreased adhesion, increased migration and caused the delocalization of alpha-actinin from plasma membrane to cytoplasm and increased expression levels of paxillin. These effects were accompanied by an increased FAK activation that physically interacted and phosphorylated paxillin and alpha-actinin. Our results are consistent with previous studies that demonstrated that paxillin and FAK overexpression, as well as their phosphorylation, were associated with low differentiation in the presence of portal vein thrombosis, along with extra-hepatic metastasis, all synonyms of high malignancy in HCC [16], [41]. However, few studies connect the expression of these proteins with HCV-dependent HCC [19], [42]. In addition, the HCV-dependent membrane to cytosol alpha-actin translocation could be the direct result of its increased phosphorylation by FAK; the increased expression of paxillin remains to be elucidated. Paxillin overexpression in HCC cells under different stimuli could be dependent on the activation of EMMPRIN(CD147)-mediated activation of FAK signaling pathways [43], [44]. Reversal effects of FAK silencing emphasize the role of this kinase enzyme in HCV-dependent effects on hepatocyte-like cells. Therefore, HCV seems to be implicated in inducing FAK activity, which in turn affects adhesiveness and the migratory ability of infected cells interfering with cytoskeleton re-organization.

Recently, it has been suggested that transformed hepatocytes, bearing HCV, are particularly active in stimulating neighboring cells, particularly HSCs, and in exerting paracrine effects [37], [45]. Our results demonstrated that increased HCV-dependent FAK activation may be a link among fibrosis, tumorigenesis and tumor progression by inducing ECM deregulation. Our idea is supported by the fact that FAK alterations may characterize both fibrosis and malignancy. In fact, the activation of FAK in Huh7.5.1 cells is associated with an increased cell proliferation, expression of alpha-SMA and HA release. Activation of HSCs may be cytokine-driven. During HCV infection, hepatocyte damage causes activation of Kupffer cells and consequent release of several different cytokines including TNF-alpha. This systemic inflammation coupled with the HCV-dependent enhancement of TNF-alpha production in hepatocyte, may be responsible of fibrogenesis and eventually hepatocarcinogenesis [46], [47]. This hypothesis is supported by previous studies demonstrating that circulating levels of TNF-alpha increase during HCV infection, and that this elevation correlates with severity of fibrosis and tissue-damage [48]. Accordingly, we observed that HCV infection induced TNF-alpha production and release by hepatocytes suggesting that TNF-alpha should be further investigated as one of the crucial circulating factors that mediate HSCs activation during HCV infection [49]. Furthermore, we found that the silencing of FAK significantly enhances the HCV-dependent apoptosis in LX-2, improving their ability to spontaneously resolve fibrogenesis.

In conclusion, we demonstrated that HCV infection, besides modifying the ECM network in hepatoma cells, stimulates mesenchymal cell proliferation and activation; in fact, the HCV infection causes the inappropriate indirect induction of HSCs, the main player in liver fibrosis occurrence, which in turn, greatly enhances the risk of subsequent cancer development.

Control of focal adhesion may represent not only a strategy to manage liver fibrosis, but also a novel therapeutic strategy for the control of HCC progression. This connection provides a new perspective on the investigation of biomolecular markers of HCC development and progression, as well as a new indication for anti-fibrotic and anti-oncogenic therapeutic targets.

## Materials and Methods

### Cell Culture

Huh7.5.1 human hepatoma cells and human HSC LX-2 cells were grown in Dulbecco’s modified Eagle’s medium (DMEM); supplemented with 2 mmol/l L-glutamine, 100 units of penicillin per ml, 100 mg of streptomycin per ml, and 10% fetal bovine serum (Gibco, Milan, Italy) at 37°C in 5% CO_2_.

### JFH1-derived ccHCV and Cell Infection

JFH1-derived ccHCV (MTA agreement number 901) was generated and used according to Kato and colleagues [24]. Huh7.5.1 were infected with JFH1-derived virus (MOI = 0.1) 1 day after plating [50]. After 5 days cells were harvested and 2,5×10^5^ JFH1-derived ccHCV/Huh7 5.1 cells were re-plated in 60 mm dishes. Medium collected from uninfected and HCV-infected Huh7.5.1 was treated with UV (254 nm for 2 min) to obtain about 90% virus inactivation before to the LX-2 treatment.

#### Reverse Transcription (RT) PCR

Total RNA was extracted from cell culture supernatant using Trizol reagent (Invitrogen San Diego, CA). Five micrograms of total RNA was reverse-transcribed with the Super Script III First-Strand Synthesis System RT-PCR, according to the manufacturer’s instructions (Invitrogen). The PCR primers used to amplify the 5′-untranslated region (5′UTR), were as follows: 5′-TCTGCGGAACCGGTGAGTAC-3′ and 5′-TCAGGCAGTACCACAAGGCC-3′.

#### siRNA transfection

FAK siRNA: (5¢-GCGAUUAUAUGUUAGAGAUAGUU-3¢) and scramble siRNA: (5¢-GCAAGCTGACCCTGAAGTTCA-3¢) were transfected at increasing concentrations from 20 to 80 nM, using Lipofectamin 2000 reagent (Invitrogen), according to the manufacturer’s protocols. Silencing of FAK protein expression was confirmed by Western blot.

#### Cell proliferation test

Cells were cultured in a 96-well plate at the indicated timepoints, and then analyzed by a colorimetric immunoassay for the quantification of BrdU incorporation to measure DNA synthesis that provides an estimation of cell proliferation rate. To this purpose we used a Cell Proliferation ELISA BrdU, kit, according to the suggested protocol (Roche, Indianapolis, IN). Quantitative data of the analysis of BrdU incorporation was converted in units of induction. Cell growth rate was evaluate as previously described [51].

#### Cell adhesion test

HCV-infected cells were grown to 70−80% confluence, washed, trypsinised, and viable cells counted under a light microscope, after Trypan Blue exclusion. Cells were then resuspended in serum-free medium, seeded in 24-well plates (3×10^5^ cells per well) and incubated for 30 or 180 min at 37°C, 5% CO_2_. Thereafter, plates were washed with PBS buffer and adherent cells were trypsinised and counted, as described above.

#### Cell migration test

A migration test was performed in a BD Biocoat Invasion Chamber with 8-µm pore size (BD Biosciences, Milan, Italy). 3×10^5^ cells were plated in the upper part of the chamber and incubated for 6 and 24 hrs in serum-free conditions, at 37 C, 5% CO_2_. The migrated cells, adhering to the lower surface of the membrane, were instead fixed with 95% ethanol, stained with Giemsa (Sigma-Aldrich Inc., St. Louis, MO) and counted under a light microscope.

### Soft Agar Assay

Soft agar assay was performed to evaluate anchorage-independent growth of uninfected and HCV-infected Huh7.5.1 cells in the presence or absence of siFAK. Briefly, 5×10^3^ Ctrl and HCV cells (eventually pre-treated for 24 hrs with siFAK as already described) were cultured in soft agar medium (10% FBS) for 28 days. Following this incubation period, colonies were stained with 0.005% Crystal Violet and counted. Colonies >0.15 mm in size were considered relevant and the colony forming efficiency was determined by the percentage of colonies per plate.

### Invasion Assay

For the invasion assay 1×10^5^ LX-2 in 300 µL serum-free medium were added into the upper chamber of the Cell Invasion Assay Kit (Millipore Corporation, Billerica, MA). Then 500 µL of NM, CM, scramble-NM, siFAK-NM, scramble-CM or siFAKCM were added to the lower chamber. The amount of invasive cells in the lower chamber was analysed after 24 hrs as recommended by the manufacturer protocol.

### Immunoprecipitation and Western Blot Analysis

Briefly, cells were collected and lysed with Ripa buffer containing 50 mM Tris (pH 7.5), 250 mM NaCl, 0.1% Nonidet P-40, 1 mM EDTA, 2 mM phenylmethilsulfonyl fluoride (PMSF), 1 µg/ml aprotinin, 1 µg/ml of leupeptin and phosphatase inhibitors. 500 µg of proteins were immunoprecipitated with specific primary antibodies for 4–5 hours and thus, the samples were incubated with A agarose (Santa Cruz Biotech, Santa Cruz, CA) for 1 hours at 4°C. Three washes were performed and the samples were solubilized and prepared to be electrophoresed on SDS-PAGE as already described [51]. Proteins were transferred to PVDF membrane and treated with specific antibodies overnight at 4°C. Then filters were washed four times and newly incubated with peroxidase-coupled secondary antibodies for 1 hour, at room temperature. After incubation the sheets were visualized by ECL kits (Amersham Pharmacia Biotech, Piscataway, NJ).

### Immunofluorescence Staining and Confocal Microscopy

Cells were plated onto 4-well chamber slide (Nunc; Naperville, IL), fixed −20°C methanol/acetone (1∶1) for 10 minutes, incubated with the different primary antibodies and revealed with two secondary antibodies (FITC- and TRIC-conjugated). After washing in PBS, nuclei were counterstained with 4¢,6-diamidine-2¢-phenylindole dihydrochloride (DAPI; Roche, Mannheim, Germany) for 2 min. After extensive washing, the samples were mounted with PBS/glycerol (1∶1) and covered by a coverslip. Images were immediately acquired by confocal laser microscopy. Confocal imaging was performed on Olympus fluoview FV1000 confocal microscope equipped with FV10-ASW version 1.6 software, multi-line argon (T = 458–488 and 514 nm) and 2X helium/neon (T = 543 and 633 nm) lasers with 60X, 1.42 N.A. oil immersion objective.

### Antibodies

Antibodies used: anti-core protein monoclonal antibody (Affinity BioReagents, Inc., Golden, CO); anti-NS3, anti-FAK, anti-paxillin, anti-alpha-actinin, anti-alpha-smooth muscle actin, anti-beta-actin and anti-phosphotyrosine monoclonal antibodies (Santa Cruz Biotech.); peroxidase-conjugated goat anti rabbit and anti-mouse IgG (Sigma-Aldrich Inc.); FITC-conjugated anti-mouse and TRIC-conjugated anti-rabbit (Sigma-Aldrich Inc.).

### Patients and Specimens

The study was performed on 10 archived liver tissues obtained from 6 patients with HCV-related HCC and 4 control non-cirrhotic subjects obtained from patients enrolled at the Surgical Departments, University of Rome “La Sapienza”, and at the Azienda Ospedaliero-Universitaria di Parma. Fresh samples were mount in OCT embedding compound, and freeze at −80°C. This study was approved by the ethical committee of “La Sapienza” University (Rome) according to the principles expressed in Declaration of Helsinki, and written informed consents were obtained from all recruited patients.

### ELISA Assays

LX-2 cells were treated with 50% JFH1-derived ccHCV-Huh7.5.1 medium (infective medium) and 50% complete DMEM. 24 hours later supernatants were collected and subjected to ELISA for hyaluronic acid (HA) and TNF-alpha according to the manufacturer’s protocol (R&D Systems, Minneapolis, MN).

LX-2 extracts were lysed according to the manufacturer’s instructions to perform the analysis of cleaved PARP by a kit purchased from Invitrogen.

### RNA Extraction, Reverse Transcription, and Real-time PCR Analysis

Total RNA was extracted using TRIzol reagent (Invitrogen) according to the manufacturer’s protocol. Single-stranded cDNA was obtained by reverse transcription of 1 µg of total RNA using the High Capacity cDNA Archiving Kit (Applied Biosystems, Foster City, CA). Real-time PCR reactions were carried out following the manufacturer’s protocol. mRNAs expression and the endogenous control gene ActB, were measured in each sample using TaqMan Gene Expression assays on ABI PRISM 7900 HT Sequence Detection System (Applied Biosystems), using TaqMan Universal PCR Master Mix (Applied Biosystems). Specific primers and the TaqMan MGB probes (6-FAM dye-labeled) were purchased from Applied Biosystems for detecting the TNF-alpha and alpha-SMA gene. A TaqMan human beta-actin MGB (6-FAM dye-labeled) control reagent kit was used to detect human beta-actin. For each sample, three replicates were run for each gene in a 96-well format plate. Gene expression values were determined as ΔCt (Ct_(sample)_−Ct_(GAPDH)_) and relative quantities between different samples were determined as ΔΔCt (ΔCt_(sample1)_−ΔCt_(sample2)_) [52].

### Statistical Analysis

Data are expressed as mean ± S.D. of at least three sample replicates, unless stated otherwise. All statistical comparisons were conducted using Student’s *t* test. P values less than 0.05 were considered statistically significant. *P<0.001, **P<0.01, ***P<0.05.

## Supporting Information

Figure S1 **Growth profile of uninfected Huh7.5.1 cells (Ctrl) and HCV-infected Huh7.5.1 counted at 0, 48, and 93 hrs.** Results reported in the growth curves are the mean ±SD (*bars*) of three independent experiments, each performed in duplicate. *P<0.05 *versus* Ctrl.
(TIF)

Figure S2 **Protein expression levels of paxillin and alpha-actinin 24 hrs after siRNA transfection (siFAK and scramble). Immunoblots are representative of at least four independent experiments.**
(TIF)

Figure S3 **Merge (** ***upper panels*** **) and colocalization** ***(lower panels*** **) of paxillin (** ***green*** **), and alpha-actinin (** ***red*** **) by confocal microscopy in Ctrl and HCV Huh7.5.1 cells 24 hrs after siRNA transfection.** Dapi (*blue*) was included to stain the nuclei. Magnification bar: 30 µ.
(TIF)

Figure S4 **Real-time PCR for the expression of alpha-SMA mRNA.** The histogram reports the relative quantity of alpha-SMA mRNA normalized for actin. Values between +1 and –1 indicating invariant expression, are not shown. *P≤0.001.
(TIF)
