# Supplementary figures and images for: Focal Adhesion Kinase (FAK) Mediates the Induction of Pro-Oncogenic and Fibrogenic Phenotypes in Hepatitis C Virus (HCV)-Infected Cells
Source: PLoS One. 2012 Aug 28;7(8):e44147. doi: 10.1371/journal.pone.0044147 (PMC3429423; doi:10.1371/journal.pone.0044147)

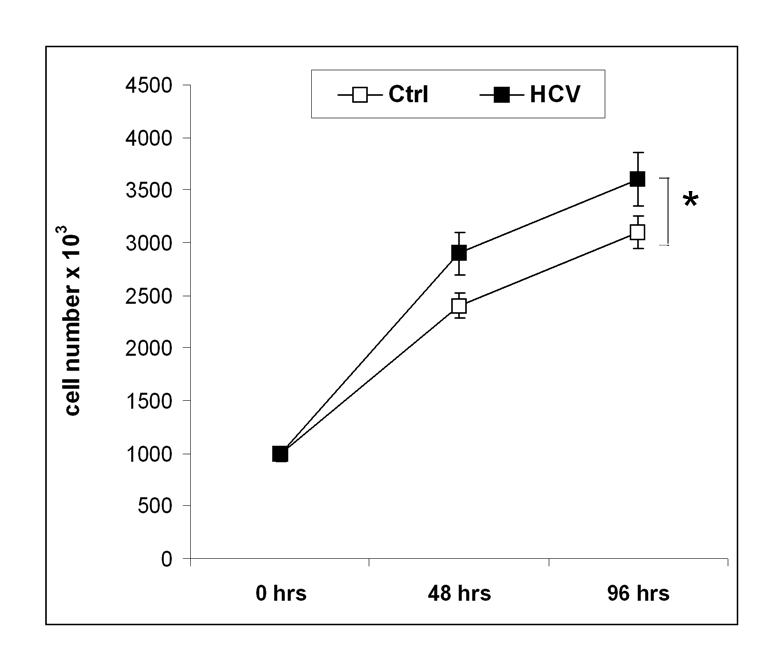

Supplement: Figure S1 — Growth profile of uninfected Huh7.5.1 cells (Ctrl) and HCV-infected Huh7.5.1 counted at 0, 48, and 93 hrs. Results reported in the growth curves are the mean ±SD (bars) of three independent experiments, each performed in duplicate. *P<0.05 versus Ctrl. (TIF) [file pone.0044147.s001.tif]

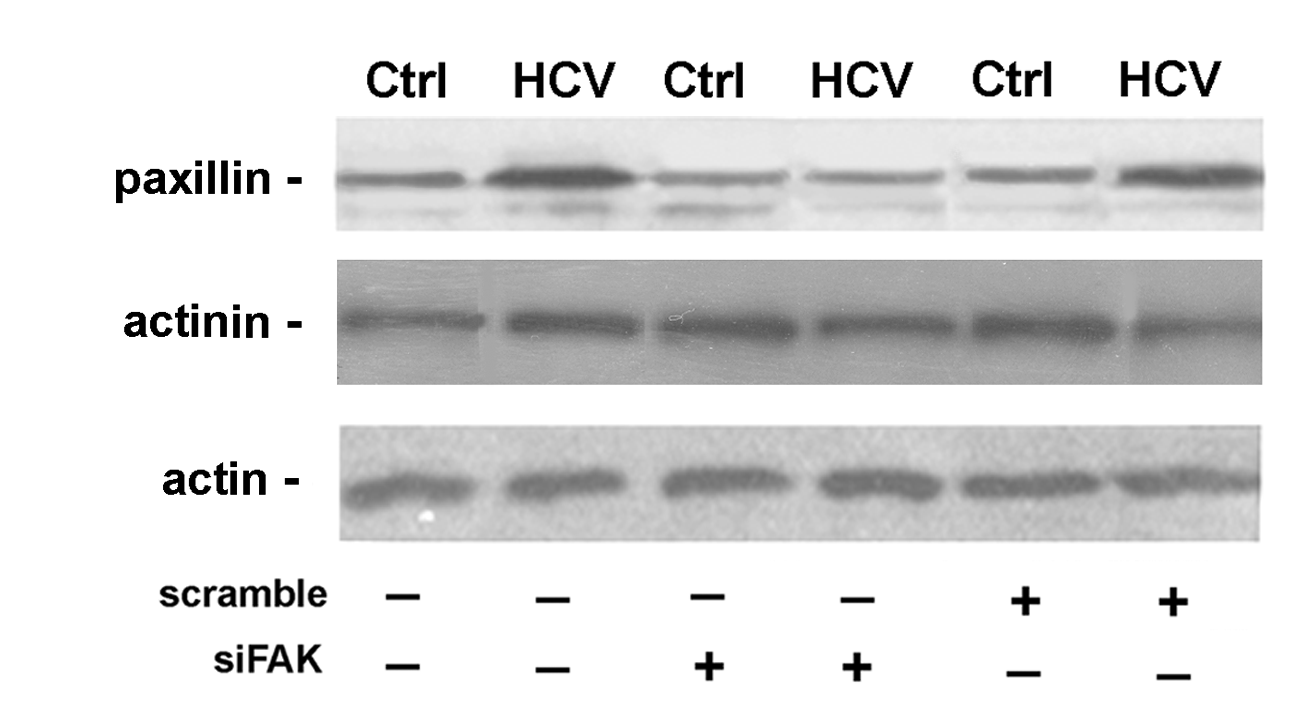

Supplement: Figure S2 — Protein expression levels of paxillin and alpha-actinin 24 hrs after siRNA transfection (siFAK and scramble). Immunoblots are representative of at least four independent experiments. (TIF) [file pone.0044147.s002.tif]

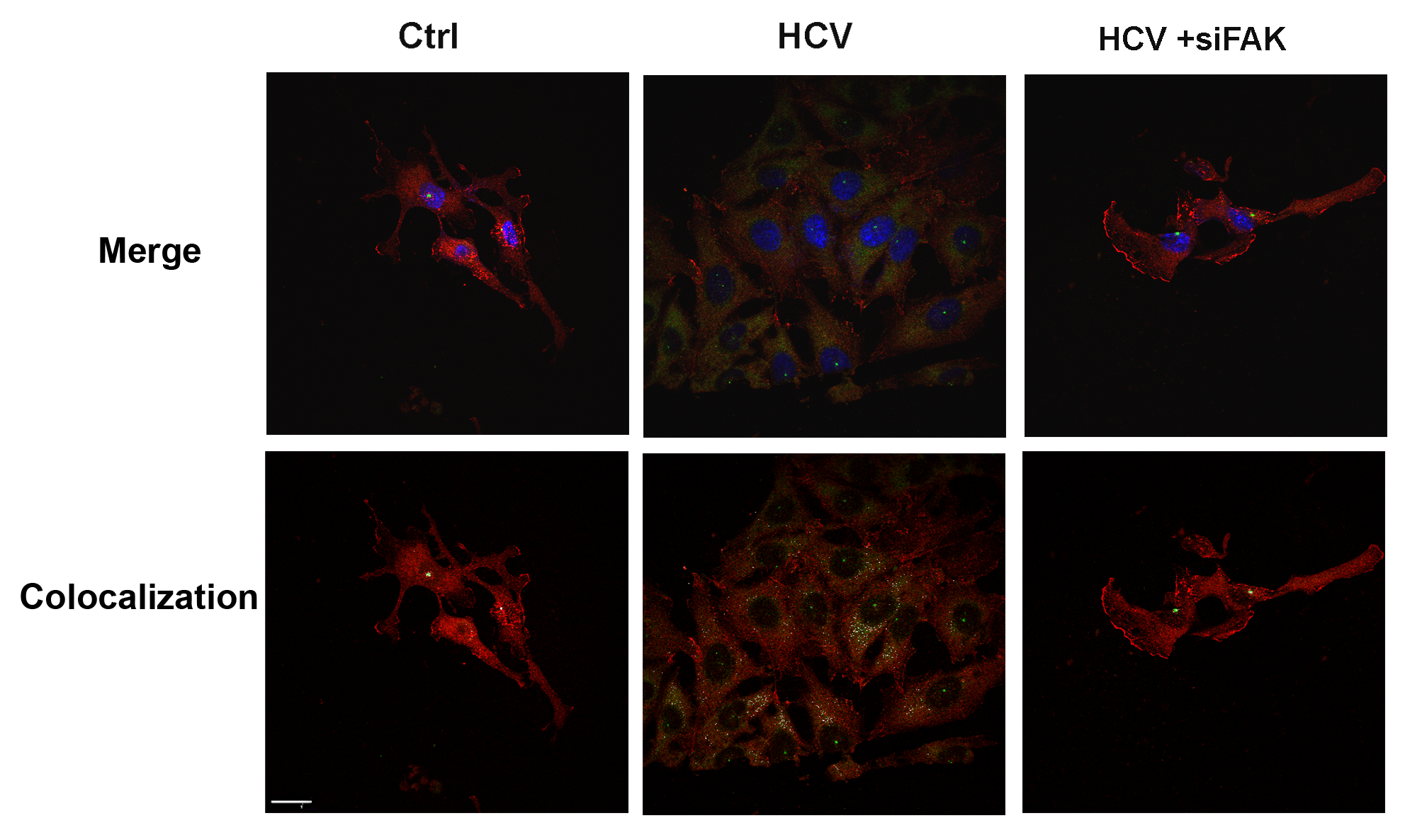

Supplement: Figure S3 — Merge ( upper panels ) and colocalization (lower panels ) of paxillin ( green ), and alpha-actinin ( red ) by confocal microscopy in Ctrl and HCV Huh7.5.1 cells 24 hrs after siRNA transfection. Dapi (blue) was included to stain the nuclei. Magnification bar: 30 µ. (TIF) [file pone.0044147.s003.tif]

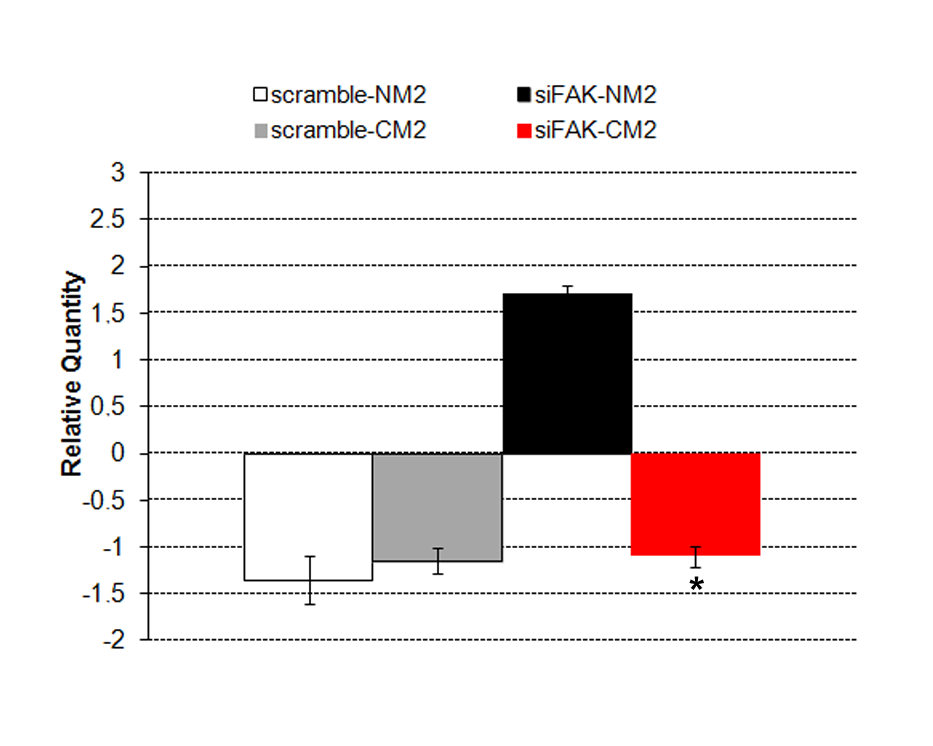

Supplement: Figure S4 — Real-time PCR for the expression of alpha-SMA mRNA. The histogram reports the relative quantity of alpha-SMA mRNA normalized for actin. Values between +1 and –1 indicating invariant expression, are not shown. *P≤0.001. (TIF) [file pone.0044147.s004.tif]
